# Palmitoylation of the human cytomegalovirus tegument protein pp28 facilitates virus release

**DOI:** 10.1371/journal.ppat.1013894

**Published:** 2026-01-22

**Authors:** Jae Bong Lee, Naeok Koo, Ji Min Park, Jun-Young Seo

**Affiliations:** 1 Department of Biomedical Sciences, Yonsei University College of Medicine, Seoul, Republic of Korea; 2 Graduate School of Medical Science, Brain Korea 21 Project, Yonsei University College of Medicine, Seoul, Republic of Korea; The University of Alabama at Birmingham Department of Pediatrics, UNITED STATES OF AMERICA

## Abstract

Tegument proteins of human cytomegalovirus (HCMV) play essential roles in viral assembly, coordinating interactions among capsids, membranes, and host-derived components. pp28 (UL99), a dominant tegument protein expressed during late infection, is essential for cytoplasmic envelopment and proper trafficking to the viral assembly compartment (vAC). Here, we identify a critical role for palmitoylation in pp28 function. Using site-directed mutagenesis and acyl-resin assisted capture (acyl-RAC) assays, we show that palmitoylation occurs at conserved cysteine residues (Cys6, Cys10, Cys11) near the N-terminus. Disruption of these residues impairs pp28 stability, alters its subcellular localization, and reduces the release of infectious virions without affecting intracellular viral replication. Confocal imaging and proteasome inhibition experiments reveal that palmitoylation-deficient pp28 is more susceptible to degradation and fails to accumulate at ERGIC-derived membranes. Consistent with these findings, recombinant HCMVs encoding pp28 mutants impaired in palmitoylation exhibit reduced extracellular viral titers. These results define palmitoylation as a key modification of pp28 that ensures proper compartmental targeting and virion maturation, underscoring a broader role for tegument lipidation in herpesvirus assembly and egress.

## Author summary

Human cytomegalovirus (HCMV) is a globally prevalent virus that poses a serious threat to individuals with weakened immune systems. To complete its life cycle, HCMV relies on tegument proteins, which help coordinate the assembly and release of new viral particles. In this study we show that the viral tegument protein pp28 requires palmitoylation for its stability and proper localization in infected cells. When this modification is disrupted, pp28 becomes unstable and fails to reach the compartments necessary for virus assembly, leading to a significant reduction in the production of infectious virus. Using genetic and biochemical approaches, we identified specific amino acids essential for this modification and confirmed its functional importance during infection. These findings highlight a previously underappreciated role for tegument proteins in viral egress and suggest that controlling protein lipidation could be a promising strategy to disrupt the production of infectious cytomegalovirus particles.

## Introduction

Human cytomegalovirus (HCMV), a member of the β-herpesvirus family, infects over 60% of the global population and is typically asymptomatic in immunocompetent individuals [1]. However, in immunocompromised patients such as those with AIDS or undergoing organ transplantation, HCMV can cause severe disease manifestations including retinitis, pneumonitis and graft rejection [2,3]. The mature HCMV virion consists of four structural components: a double-stranded DNA genome, an icosahedral capsid, a tegument layer located between the capsid and the envelope, and a host-derived lipid envelope embedded with viral glycoproteins [4,5]. Tegument proteins are multifunctional and contribute to various aspects of the viral life cycle, including virion assembly, intracellular trafficking, immune modulation, and host-pathogen interactions [6,7].

pp28, encoded by the UL99 open reading frame, is a true late tegument protein and one of the most abundant components of the tegument layer [8]. It plays a critical role in the cytoplasmic envelopment of capsids during virion maturation and is required for the formation of mature virions [9–11]. When expressed in the absence of infection, pp28 localizes to the endoplasmic reticulum (ER)-Golgi intermediate compartment (ERGIC), a membrane trafficking hub between the ER and the Golgi apparatus [12]. During infection, pp28 accumulates in the viral assembly compartment (vAC) [13]. This protein undergoes multiple post-translational modifications including myristoylation at glycine 2 and phosphorylation at serine and threonine residues which are required for membrane targeting and trafficking to the vAC, respectively [12,14]. However, the functional significance of pp28 palmitoylation remains poorly understood.

Palmitoylation (a type of S-acylation) is a reversible post-translational lipid modification in which a 16-carbon palmitic acid is covalently attached to cysteine residues via a thioester bond [15,16]. This modification increases the hydrophobicity of proteins and facilitates their membrane association, protein-protein interactions, intracellular trafficking, and stability [17]. Acylated cysteine residues are frequently located near transmembrane domains, where they enhance membrane anchoring. In the context of viral replication, palmitoylation of viral proteins has been shown to influence virion assembly, envelope formation, and infectivity [18–20]. Although most palmitoylated viral proteins identified to date are envelope glycoproteins [21,22], several tegument proteins in other herpesviruses such as HSV-1 UL11 have also been reported to undergo palmitoylation which implies that this modification may play a broader role in tegument function and viral maturation [23].

In this study we investigated the palmitoylation of HCMV pp28 and its role during infection. Site-directed mutagenesis and an acyl-resin assisted capture (Acyl-RAC) analysis revealed that three cysteine residues at amino acid positions 6, 10, and 11 in the N-terminal region of pp28 are critical for its palmitoylation. We further show that palmitoylation prevents proteasomal degradation of pp28 and is required for its proper localization to the ERGIC. Importantly, when these residues were substituted with alanine in the context of viral infection, the release of infectious virions was significantly impaired, while intracellular viral replication remained unaffected. Our findings reveal a previously uncharacterized mechanism of HCMV maturation and highlight palmitoylation as a key modulator of tegument protein function.

## Results

### Palmitoylation of pp28 is essential for proper protein function

**Fig 1:**
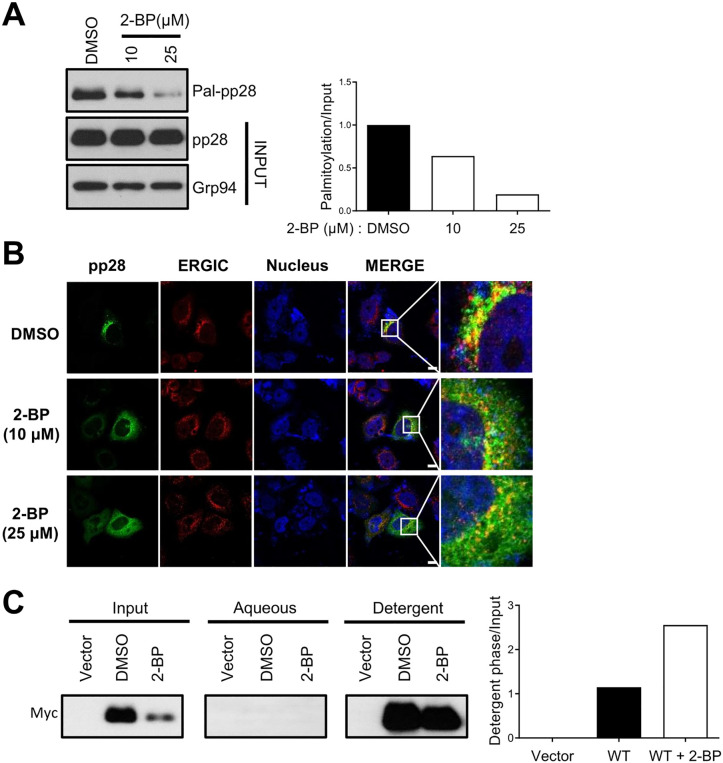
Palmitoylation of pp28 and its inhibition disrupts the protein’s subcellular localization. **(A)** Acyl-resin–assisted capture (Acyl-RAC) assay was used to demonstrate pp28 palmitoylation. 293T cells transiently expressing pp28 were treated with either DMSO (control) or increasing concentrations (10 µM and 25 µM) of 2-bromopalmitate (2-BP), a palmitoylation inhibitor for 24 h. Palmitoylated pp28 (Pal-pp28) was captured using the Badrilla CAPTUREome S-Palmitoylated Protein Kit and detected by immunoblotting with an anti-pp28 antibody. A dose-dependent reduction in palmitoylation was observed with 2-BP treatment. The input panel shows total pp28 and Grp94 as a loading control. Bar graphs represent the relative palmitoylation levels normalized to input pp28. **(B)** Immunofluorescence analysis of 293T cells expressing pp28, treated with DMSO or 2-BP (10 µM or 25 µM) for 24 h. Cells were stained for pp28 (green), the ERGIC marker ERGIC-53 (red), and nuclei (DAPI, blue). In DMSO-treated cells, pp28 localized predominantly to the perinuclear region, overlapping with the ERGIC marker. Following 2-BP treatment, pp28 localization became progressively diffuse and cytoplasmic. Scale bars, 10 µm. **(C)** HEK293T cells were transiently transfected with wild-type pp28-myc and treated with DMSO or 2-BP (50 µM) for 24 h. Cells were extracted in 1% Triton X-114 at 4°C, and phase separation was induced at 30°C using a sucrose cushion. The detergent and aqueous fractions were collected and analyzed by SDS–PAGE followed by immunoblotting with an anti-myc antibody. Bar graphs represent the relative quantification of pp28 collected in the detergent phase, normalized to input pp28.

Previous studies have reported that pp28 is palmitoylated, although the functional consequences of this modification are not yet fully understood [22]. To initially verify this post-translational modification in our system, we performed an Acyl-RAC assay [24] using 293T cells transiently expressing pp28. Our results confirmed that pp28 is indeed palmitoylated (Fig 1A). Furthermore, treating the cells with the palmitoylation inhibitor 2-bromopalmitate (2-BP) [25] dose-dependently decreased pp28 palmitoylation levels (Fig 1A), confirming the specificity of this lipid modification. To examine whether palmitoylation governs the subcellular distribution of pp28, 293T cells transiently expressing pp28 were treated with increasing concentrations of 2-BP, and the localization of the protein was assessed. In untreated cells, pp28 exhibited a perinuclear localization pattern, consistent with its known accumulation in the ERGIC. However, 2-BP treatment progressively disrupted pp28 localization in a dose-dependent manner, resulting in a more diffuse cytoplasmic distribution (Fig 1B). These findings indicate that palmitoylation is essential for the proper subcellular localization of pp28 and may be crucial for its membrane association and trafficking during viral assembly. To directly determine whether palmitoylation of pp28 accounts for its membrane association, we compared the solubility of pp28 in the detergent TX114 with and without 2-BP treatment. Biochemical fractionation revealed that 2-BP–treated pp28 remained predominantly in the detergent phase to a similar extent as untreated pp28 (Fig 1C). The results indicate that palmitoylation of pp28 does not affect its membrane association.

### Prediction of palmitoylation sites on pp28

**Fig 2:**
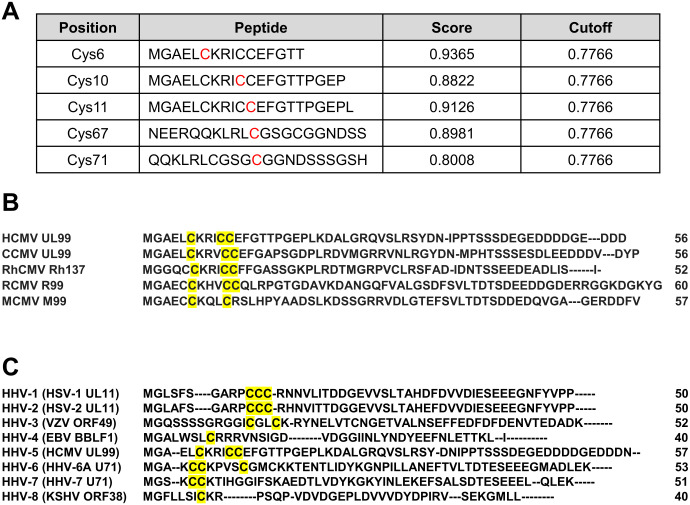
N-terminal cysteines of pp28 are conserved palmitoylation sites in herpesviruses. **(A)** The amino acid sequence of pp28 (UL99) was analyzed using CSS-Palm 4.0 to predict S-palmitoylation sites. The algorithm identified five cysteine residues above the high-stringency cutoff (0.7766). Peptide sequences show the local motif, with the predicted cysteines highlighted in red. **(B)** Multiple sequence alignment of the N-terminal region of pp28 (UL99) orthologs from representative mammalian cytomegaloviruses (CMVs), with UniProt accession numbers in parentheses: human CMV (HCMV) UL99/pp28 (P13200), chimpanzee CMV (CCMV) UL99 (Q8QS01), rhesus CMV (RhCMV) Rh137 (Q2FAH3), rat CMV (RCMV) R99 (Q9DWA1), and murine CMV (MCMV) M99 (D3XDS4). The alignment, performed using Clustal Omega, highlights conserved cysteine residues in the N-terminal region in yellow, indicating strong evolutionary conservation among CMVs. **(C)** Alignment of the N-terminal region of pp28 orthologs from representative human herpesviruses (HHVs), with UniProt accession numbers in parentheses: herpes simplex virus (HSV)-1 UL11 (P04289), HSV-2 UL11 (P13294), varicella-zoster virus (VZV) ORF49 (P09297), Epstein–Barr virus (EBV) BBLF1 (P0CK51), HCMV UL99/pp28 (P13200), HHV-6A U71 (P24448), HHV-7 U71 (P52358), and Kaposi sarcoma–associated herpesvirus (KSHV) ORF38 (F5HHY1).

Palmitoylation typically occurs through the covalent attachment of palmitic acid to cysteine residues via a thioester bond. pp28 contains five cysteine residues at amino acid positions 6, 10, 11, 67, and 71. To predict palmitoylation sites, we analyzed the pp28 amino acid sequences using the prediction algorithm CSS-Palm 4.0 [26]. This analysis revealed that the N-terminal cysteines (Cys6, Cys10, and Cys11) were strongly predicted to be palmitoylation sites, whereas the C-terminal cysteine at position 71 exhibited lower probabilities of palmitoylation (Fig 2A). Next, to determine whether the predicted N-terminal cysteine residues are evolutionarily conserved, we performed multiple sequence alignment of pp28 orthologs from cytomegaloviruses (CMVs) across different species (Fig 2B) and from representative members of the herpesvirus family. The N-terminal cysteine residues, particularly at positions 6 and 10, were highly conserved across CMV strains and other herpesviruses (Fig 2C). This degree of conservation suggests that these residues are functionally important and may represent conserved palmitoylation motifs. Collectively, these data indicate that the N-terminal cysteine residues of pp28 are likely sites of palmitoylation and are evolutionarily conserved among CMVs and herpesviruses, thereby supporting the hypothesis that these residues play a critical role in pp28 function.

### N-terminal cysteines Cys6, Cys10, and Cys11 are essential for pp28 palmitoylation

**Fig 3:**
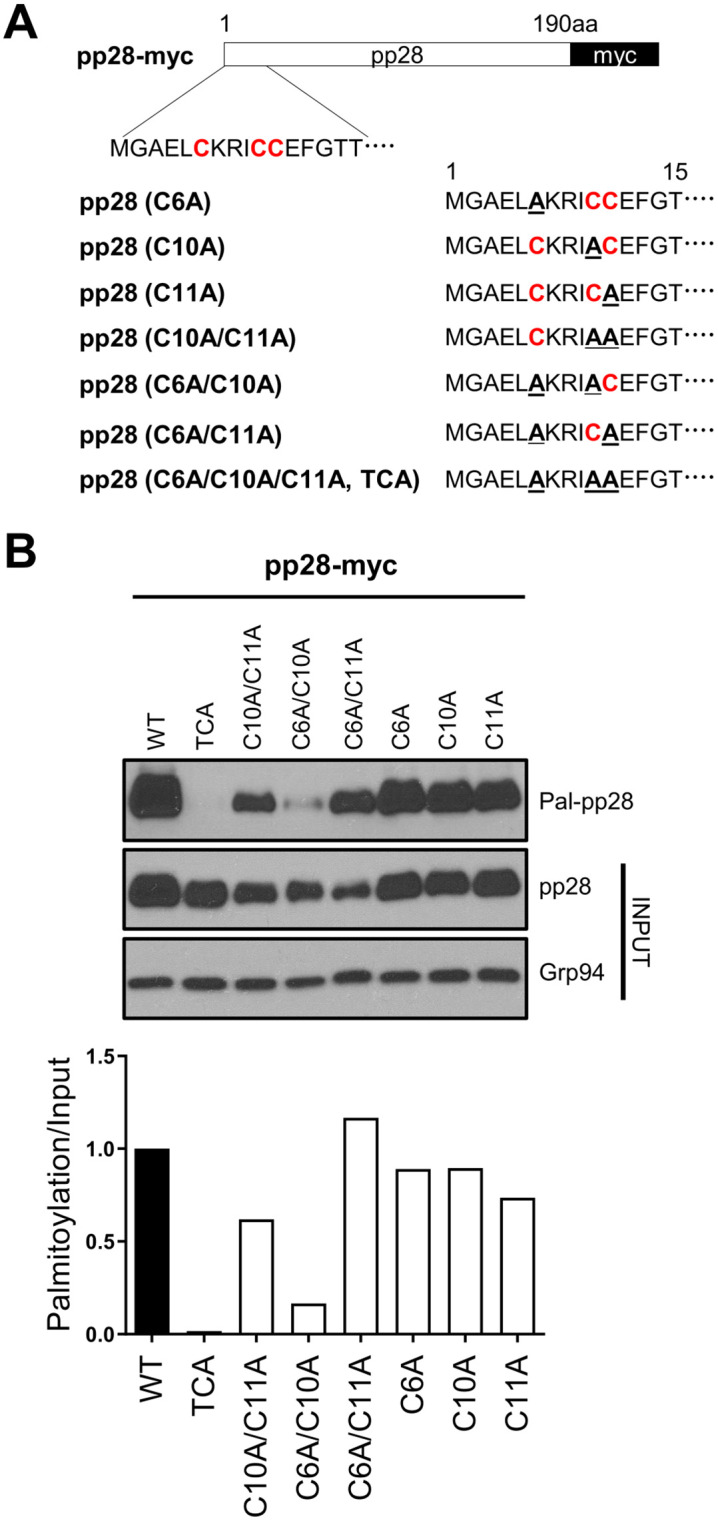
pp28 is palmitoylated at three N-terminal cysteine residues. **(A)** Schematic of pp28-myc constructs and cysteine-to-alanine substitution mutants. The three N-terminal cysteine residues (C6, C10, and C11) were individually or combinatorially substituted with alanine. The triple mutant (C6A/C10A/C11A) is referred to as TCA. **(B)** Acyl-RAC assay in 293T cells expressing wild-type or mutant forms of pp28. Palmitoylated pp28 (Pal-pp28) was detected by immunoblotting. Substitution of individual cysteine residues led to a partial reduction in palmitoylation, while combined mutations resulted in progressively diminished signals. The TCA mutant completely abolished pp28 palmitoylation. Input panels show total pp28 and Grp94 as a loading control. Bar graphs represent the relative palmitoylation levels normalized to input pp28.

To validate the predicted palmitoylation sites in pp28, we generated a series of cysteine-to-alanine substitution mutants targeting the three N-terminal cysteines (Cys6, Cys10, and Cys11) in the pp28 sequence (Fig 3A). These included single, double, and a triple mutant to evaluate the individual and combined contribution of each residue to palmitoylation. Acyl-RAC assays were performed in 293T cells transiently expressing wild-type or mutant forms of pp28. Substitution of each individual N-terminal cysteine resulted in a moderate reduction in palmitoylation, whereas double mutations led to a more pronounced decrease. Notably, the triple mutant (TCA, C6A/C10A/C11A) in which all three N-terminal cysteine residues (Cys6, Cys10, and Cys11) were substituted with alanine completely abolished pp28 palmitoylation, indicating that these three residues are collectively required for the modification (Fig 3B). To exclude a possibility that cysteine residues in the C-terminal region contribute to pp28 palmitoylation, we also generated alanine substitution mutants at positions 67 and 71. Acyl-RAC analysis revealed that palmitoylation levels in the C67A and C67A/C71A mutants were comparable to those in wild-type pp28, indicating that these residues do not participate in the modification (S1 Fig). These results demonstrate that pp28 is palmitoylated specifically at the N-terminal cysteines—Cys6, Cys10, and Cys11—and highlight the importance of empirical validation in identifying functionally relevant palmitoylation sites, even when bioinformatic predictions are available.

### Palmitoylation governs pp28 stability and subcellular localization

**Fig 4:**
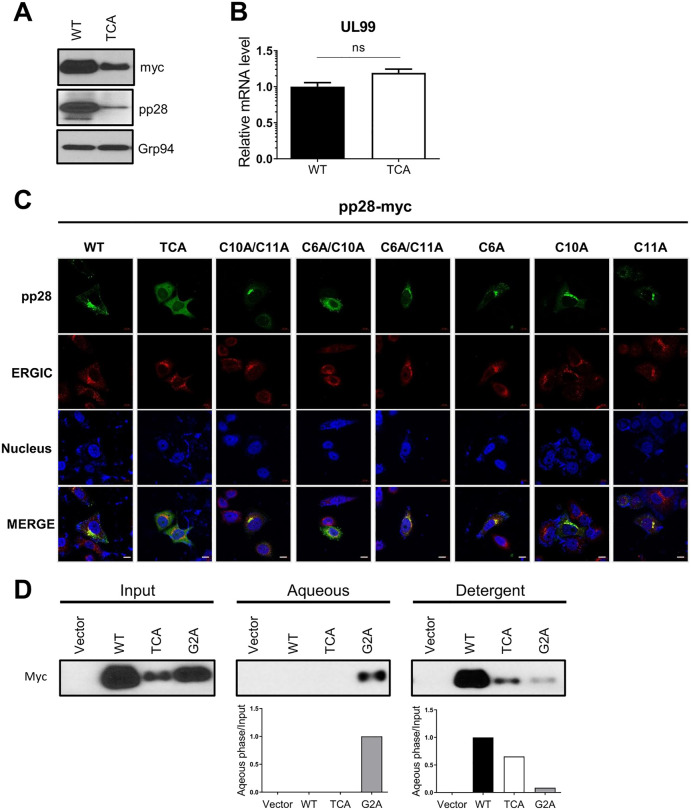
pp28 palmitoylation determines its stability and subcellular localization, not membrane association. **(A)** Immunoblot analysis of 293T cells transiently expressing wild-type pp28 or the palmitoylation-deficient mutant (TCA). **(B)** RT-qPCR analysis of pp28 mRNA in cells transfected with wild-type or TCA constructs. Relative pp28 mRNA levels were normalized to β-actin mRNA (*n* = 2 in triplicate). Transcript levels were comparable, indicating that reduced protein levels are not due to differences in mRNA expression. Data are presented as means ± SEM. Statistical analysis was performed using a *t*-test. **(C)** Confocal immunofluorescence analysis of 293T cells expressing pp28-myc mutants. Cells were stained for pp28 (green), ERGIC-53 (red), and nuclei (blue, DAPI). Scale bars, 10 μm. **(D)** HEK293T cells were transiently transfected with the indicated pp28 constructs and extracted in 1% Triton X-114 at 4°C. Phase separation was induced at 30°C using a sucrose cushion, and detergent and aqueous fractions were analyzed by SDS–PAGE and immunoblotting with an anti-myc antibody. Bar graphs represent the relative quantification of pp28 collected in the detergent phase, normalized to input pp28.

Palmitoylation has been implicated in the regulation of both protein stability and subcellular localization [17]. To determine whether this modification influences pp28 stability, we compared protein expression levels between wild-type pp28 and a palmitoylation-deficient mutant (TCA). Immunoblot analysis revealed a marked reduction in the steady-state protein level of the TCA mutant compared to wild-type pp28 following transient transfection in 293T cells (Fig 4A). To determine whether this reduction was due to altered transcription, we performed quantitative real-time PCR (qRT-PCR) analysis. No significant difference in pp28 mRNA levels was observed between the wild-type and TCA constructs, indicating that the reduction in protein level occurs post-transcriptionally (Fig 4B). These findings suggest that palmitoylation contributes to the stability of pp28, potentially by protecting it from degradation. We next investigated whether palmitoylation at the three N-terminal cysteines also affects the subcellular localization of pp28. Confocal immunofluorescence analysis of 293T cells expressing myc-tagged pp28 constructs showed that wild-type pp28 localized predominantly to the perinuclear region and exhibited strong colocalization with ERGIC-53, a marker of the ERGIC [27] (Fig 4C). In contrast, the TCA mutant displayed diffuse cytoplasmic localization with minimal ERGIC overlap, indicating disrupted subcellular targeting. Among the double mutants, C6A/C10A showed a pronounced defect in ERGIC localization, while C6A/C11A and C10A/C11A exhibited more modest changes. Single mutants (C6A, C10A, and C11A) largely retained a wild-type–like localization pattern, although subtle deviations in their perinuclear enrichment were observed. The results indicate that at least two of the three N-terminal cysteines are necessary for the subcellular localization of pp28. It has been previously reported that myristoylation, another lipid modification of pp28, is required for both its membrane association and localization [12]. To examine whether palmitoylation is also necessary for these processes, we compared the solubilities of wild-type pp28, the TCA mutant, and a G2A mutant lacking the myristoylated glycine residue at position 2 in the detergent TX114. Despite the TCA mutant having a strong defect in intracellular targeting, biochemical phase partitioning analysis demonstrated that this mutant, similar to 2-BP–treated pp28 shown in Fig 1C, remained predominantly membrane-associated. In contrast to the G2A mutant, which localizes predominantly to the aqueous phase, both wild-type pp28 and the TCA mutant were enriched in the detergent phase at comparable levels (Fig 4D). These data clearly indicate that loss of palmitoylation does not impair the membrane association of pp28. In addition, we examined the correlation between palmitoylation and myristoylation of pp28. Treatment with 2-BP or the presence of the TCA mutant did not affect the myristoylation of pp28 when compared to the G2A mutant (S2A and S2B Fig). In contrast, the G2A mutant showed a marked reduction in palmitoylation (S2C Fig). These results indicate that pp28 myristoylation is independent of palmitoylation, whereas palmitoylation requires prior myristoylation, establishing myristoylation as a prerequisite for the acylation of pp28. Previous studies have shown that pp28 (UL99) interacts with the viral tegument protein UL94 and that this interaction is critical for efficient HCMV assembly and replication [28]. To assess whether pp28 palmitoylation influences this interaction, we examined the subcellular localization of UL94 when co-expressed with either wild-type pp28 or TCA mutant. Immunofluorescence analysis revealed that UL94 colocalized with wild-type pp28 at perinuclear ERGIC compartments, consistent with prior reports. Notably, when co-expressed with the TCA mutant, UL94 exhibited a diffuse cytoplasmic distribution similar to that of TCA pp28 (S3 Fig). These findings suggest that while palmitoylation of pp28 is not required for its physical interaction with UL94, it is essential for the proper subcellular localization of the complex. In cells expressing the TCA mutant, pp28 fails to localize to the ERGIC and consequently redirects UL94 to a diffuse cytoplasmic compartment. Together, these data demonstrate that palmitoylation of pp28 is essential for both its protein stability and proper intracellular localization, rather than its membrane association. N-terminal cysteine residues, specifically Cys6 and Cys10, are critical for ERGIC targeting, and their substitution leads to progressively impaired subcellular localization and reduced protein expression.

### Palmitoylation protects pp28 from proteasomal degradation

**Fig 5:**
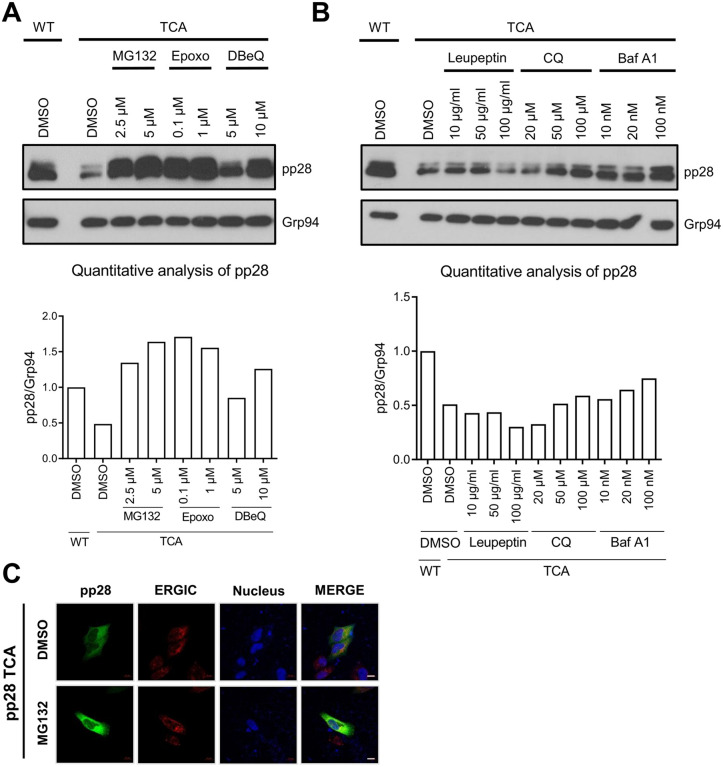
pp28 palmitoylation protects the protein from proteasomal degradation. **(A)** Immunoblot analysis of 293T cells transiently expressing TCA pp28, treated for 24 h with increasing concentrations of the proteasome inhibitors MG132, epoxomicin, or DBeQ. Grp94 was used as a loading control. The right panel shows pp28 protein band intensities quantified by densitometry and normalized to Grp94. **(B)** Immunoblot analysis of TCA-expressing cells treated for 24 h with the lysosomal inhibitors leupeptin, chloroquine, or bafilomycin A1. Grp94 served as a loading control. The right panel shows pp28 protein band intensities quantified by densitometry and normalized to Grp94. **(C)** Confocal immunofluorescence microscopy of TCA pp28-expressing 293T cells treated with DMSO or MG132. Cells were stained for pp28 (green) and ERGIC-53 (red), with nuclei counterstained with DAPI (blue). Scale bars, 10 μm.

Protein stability is often regulated through degradation pathways, predominantly involving the proteasome or the lysosome [29]. To determine which degradation system is responsible for the reduced stability of palmitoylation-deficient pp28, we treated 293T cells expressing wild-type or TCA-mutant pp28 with a panel of proteasome and lysosome inhibitors. Immunoblot analysis showed that the proteasome inhibitors MG132, epoxomicin, and VCP/p97 inhibitor DBeQ [30] restored pp28 protein levels in TCA-expressing cells in a dose-dependent manner (Fig 5A). These compounds most effectively rescued pp28 expression, indicating that proteasomal degradation is the dominant pathway for the turnover of the palmitoylation-deficient pp28 mutant. In contrast, lysosomal inhibitors such as leupeptin, chloroquine, and bafilomycin A1, had minimal effects on pp28 expression (Fig 5B). These results indicate that palmitoylation protects pp28 primarily from proteasome-mediated degradation, while the lysosomal pathway is not significantly involved. To further investigate whether proteasome inhibition could restore proper subcellular localization of pp28, we performed confocal immunofluorescence microscopy. In DMSO-treated control cells, TCA mutant pp28 was distributed throughout the cytoplasm and showed minimal colocalization with the ERGIC marker, confirming its disrupted localization. Although MG132 treatment markedly increased the steady-state level of the TCA mutant pp28, it did not restore its proper perinuclear localization. Instead, the protein remained diffusely distributed. This indicates that simply increasing protein stability is insufficient to rescue its compartment-specific targeting in the absence of palmitoylation (Fig 5C). Collectively, these findings demonstrate that palmitoylation of pp28 is required to prevent its degradation via the proteasome and to maintain both its protein stability and proper localization.

### pp28 palmitoylation at its N-terminal cysteine residues is required for its stability and localization during HCMV infection

**Fig 6:**
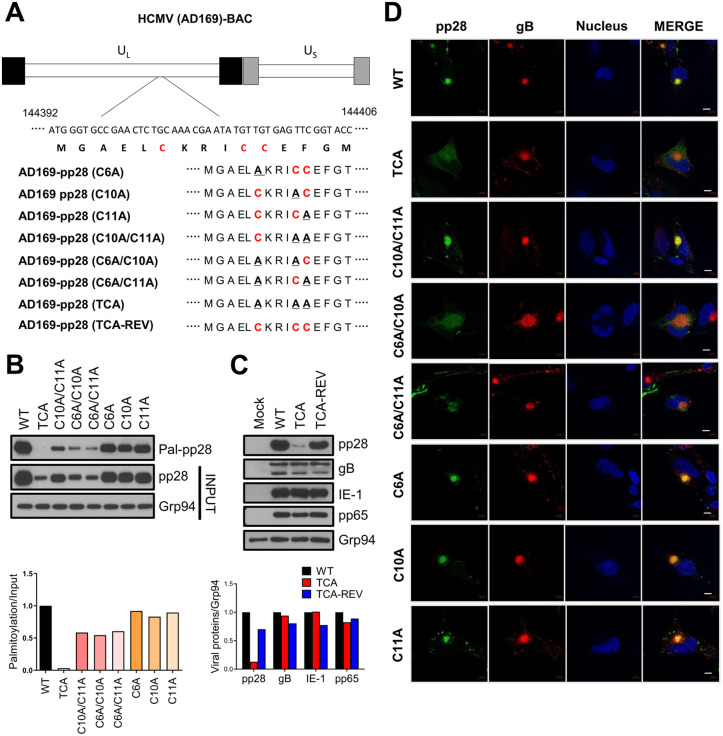
N-terminal palmitoylation of pp28 is required for its localization and stability during HCMV infection. **(A)** Schematic of cysteine-to-alanine substitutions introduced into the pp28 (UL99) coding sequence. Singl, double, and triple (TCA) mutants were generated by BAC mutagenesis. A revertant virus (TCA-REV) was constructed by restoring the original cysteines. **(B)** Acyl-RAC assay of HFFs infected with wild-type or mutant HCMV (moi = 1.0) at 3 dpi. Bar graphs represent the relative palmitoylation levels normalized to input pp28. **(C)** Immunoblot analysis of whole-cell lysates from infected HFFs at 3 dpi, probed for pp28, gB, IE1, and pp65. **(D)** Confocal immunofluorescence of HFFs at 3 dpi, stained for pp28 (green), gB (red), and nuclei (DAPI, blue). Scale bars, 10 μm.

To investigate the functional significance of pp28 palmitoylation during HCMV infection, we generated a series of recombinant viruses. Using BAC mutagenesis [31], we substituted individual or combined cysteine residues (Cys6, Cys10, and Cys11) with alanine (Fig 6A). This allowed us to create a panel of viruses, including single (C6A, C10A, and C11A), double (C6A/C10A, C6A/C11A, and C10A/C11A), and triple (TCA) mutants, as along with a revertant virus (TCA-REV) where the original cysteine residues were restored. To determine whether these mutations impair pp28 palmitoylation during infection, human fibroblasts were infected with each mutant virus, and palmitoylated pp28 was examined using Acyl-RAC. pp28 palmitoylation was variably reduced across the cysteine mutants, with the TCA mutant exhibiting markedly diminished palmitoylation (Fig 6B). These findings confirm that Cys6, Cys10, and Cys11 are the principal palmitoylation sites during HCMV infection. We then investigated whether palmitoylation affects pp28 protein stability during infection. Immunoblot analysis revealed that pp28 levels were markedly reduced in cells infected with the TCA mutant virus compared to those infected with wild-type or TCA-REV viruses. However, the expression levels of other viral proteins, including gB, IE1, and pp65, remained unchanged (Fig 6C). These findings indicate that pp28 palmitoylation specifically contributes to its stability without broadly affecting other viral protein expression. To assess how pp28 palmitoylation impacts its localization during infection, we performed immunofluorescence analysis. In cells infected with wild-type virus, pp28 accumulated in a perinuclear region and colocalized with gB-labeled viral assembly compartments (vAC). In contrast, cells infected with the TCA mutant virus showed a diffuse cytoplasmic distribution of pp28 and much less colocalization with gB (Fig 6D), indicating a targeting defect. The C6A/C10A mutant virus also showed a similar localization defect, whereas the single cysteine mutant viruses had no effect. These findings demonstrate that N-terminal palmitoylation of pp28 is required for both its subcellular stability and proper localization during HCMV infection.

### pp28 palmitoylation is essential for efficient production of extracellular HCMV virions

**Fig 7:**
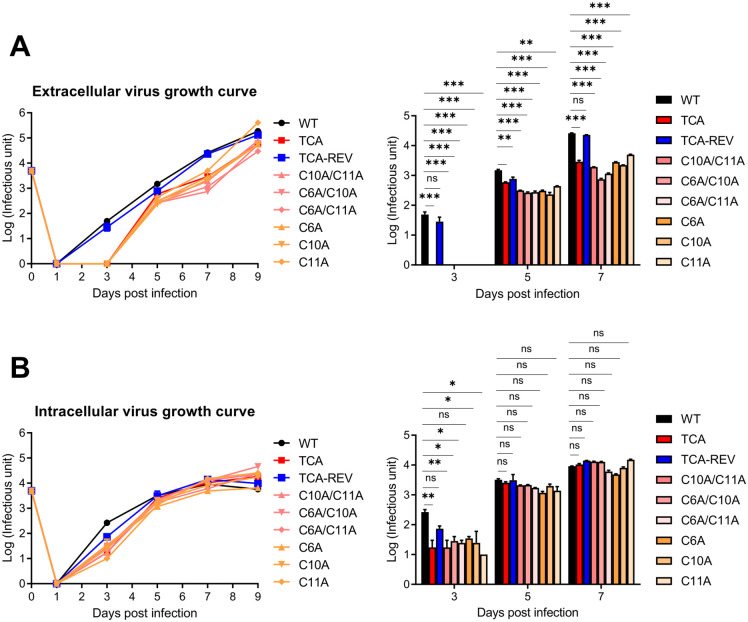
N-terminal palmitoylation of pp28 is required for efficient production of extracellular HCMV virions. Growth kinetics of recombinant HCMV mutants encoding cysteine-to-alanine substitutions in pp28. HFFs were infected at an moi of 0.05. **(A)** Extracellular and **(B)** intracellular virus titers were measured over time using IE1-based immunofluorescence titration assays. The right panels show bar graphs depicting virus titers at 3, 5, and 7 dpi. Data are presented as means ± SEM. Statistical analysis was performed using one-way ANOVA with Dunnett’s multiple-comparison test. *P < 0.05; **P < 0.01; ***P < 0.001.

To investigate the role of pp28 palmitoylation in HCMV replication and virion release, we compared virus production in HFFs infected with wild-type, pp28 palmitoylation-deficient mutants, and revertant viruses. Immunofluorescence-based titration using IE1 staining was used for rapid measurement of virus titers [32]. Quantification of extracellular virus titers revealed that pp28 palmitoylation-deficient viruses—including the triple mutant (TCA) and multiple double mutants such as C6A/C10A—exhibited delayed replication kinetics compared to the wild-type and revertant viruses. This delay was particularly evident during the early and mid-phases of infection (Fig 7A). Interestingly, the single cysteine mutant viruses also showed delayed replication kinetics, even though their localization patterns at the vAC were similar to the wild-type. This suggests that palmitoylation at these individual cysteine residues is essential for extracellular virus production, independent of its role in intracellular localization. While titers from all pp28 palmitoylation-deficient viruses increased gradually, they remained significantly lower than the wild-type, suggesting reduced efficiency or delayed timing of virion release. In contrast, intracellular virus titers were comparable across all virus strains, including the TCA mutant and the revertant (TCA-REV), indicating that intracellular viral genome replication was not significantly affected (Fig 7B). We validated the immunofluorescence-based titration method by performing parallel viral growth analyses with a conventional plaque forming assay (PFA). Consistent with the IE1 measurements, the TCA mutant showed an approximately 9-fold reduction in extracellular infectious titers compared with the wild-type and revertant viruses. However, intracellular titers were comparable across all three virus strains, the wild-type, the TCA mutant, and the revertant (TCA-REV) (S4A and S4B Fig). Furthermore, we analyzed the viral particles generated within cells infected with either the wild-type or the TCA virus. We used electron microscopy to quantify cytoplasmic non-enveloped particles (capsids and tegumented capsids) and enveloped particles (double-layered particles formed by tegumentation and secondary envelopment in the secretory pathway) late in infection, which allowed for the accumulation of a sufficient number of virions for quantification (S5A Fig). In both cells infected with the wild-type or TCA viruses, approximately 50% of the cytoplasmic viral particles were enveloped (S5B Fig). This confirms that pp28 palmitoylation-deficient viruses have no effect on the production of intracellular viruses. Next, we examined the viral genome copy numbers present in extracellular particles produced from cells infected with the wild-type, the TCA mutant, and the revertant (TCA-REV). The ratio of the viral genome copy numbers from total particles to the infectious particle numbers was comparable across all three virus strains (S5C Fig). This indicates that the TCA mutant produces a similar ratio of infectious viral particles compared to the wild-type and revertant viruses, although its release efficiency is defective. We also compared viral production of these three strains late in infection (13 dpi). Interestingly, we observed an increase of infectious intracellular virions in the TCA mutant (S5D Fig). This accumulation strongly suggests the defect lies in virion release rather than an assembly defect, confirming that the reduced extracellular virus production by the TCA mutant is due to a release defect. To ensure that the observed defects were not due to differences in viral entry, we performed entry assays using immunoblotting (S6A Fig) and fluorescence-based quantification (S6B Fig). Entry efficiency was similar among wild-type, TCA mutant, and TCA-REV viruses (S6 Fig), confirming that the replication phenotype was independent of viral entry. Taken together, these results demonstrate that while N-terminal palmitoylation of pp28 is dispensable for the formation of intracellular viral particles, it is essential for the efficient and timely release of infectious virions into the extracellular space.

## Discussion

Post-translational palmitoylation, a reversible lipid modification where palmitic acid is covalently attached to cysteine residues, is known to regulate a protein’s membrane association, subcellular trafficking, stability, and localization. While this modification is well characterized in HCMV envelope glycoproteins [21,22], its role in the tegument layer of this virus remains unknown. Studies on related herpesviruses, like HSV-1 UL11 and EBV BBLF1 [23,33], suggest that palmitoylation of tegument proteins may be a conserved mechanism that is vital for membrane targeting and particle maturation.

In this study, we identify human cytomegalovirus (HCMV) pp28, a major tegument protein, as a palmitoylated molecule. We show that palmitoylation at three conserved N-terminal cysteine residues (Cys6, Cys10, and Cys11) is essential for maintaining protein stability, proper targeting to the virus assembly compartment (vAC), and efficient production of extracellular infectious virions. Palmitoylation-deficient pp28 mutants have reduced protein stability and fail to accumulate in the ER-Golgi intermediate compartment (ERGIC) under transient expression conditions. These defects persisted even with proteasome inhibition, indicating that palmitoylation both stabilizes pp28 and plays a crucial role in determining its intracellular localization during morphogenesis.

Beyond its role in pp28 localization and stability, we explored how palmitoylation influences interactions with other tegument components. pp28 is known to interact with UL94, another tegument protein involved in virion maturation [28]. Interestingly, although this interaction is preserved without palmitoylation, the aberrant localization of both proteins in cells expressing palmitoylation-deficient pp28 highlights the spatial dependency of tegument–tegument interactions. This suggests that palmitoylation contributes to the positioning of individual proteins as well as to the structural integrity of multiprotein tegument assembly.

Our data also demonstrate that pp28 palmitoylation is required for proper subcellular localization and virion egress. Recombinant viruses with the palmitoylation-deficient pp28 mutant (TCA) produced intracellular viral particles at levels comparable to the wild-type, but had significantly reduced extracellular infectious titers. These results suggest that the accurate subcellular targeting of pp28, which is mediated by palmitoylation, is required for secondary envelopment and subsequent steps involved in the release of mature virions. Furthermore, recombinant viruses with the single cysteine mutants (C6A, C10A, and C11A) also had reduced extracellular infectious titers, despite having similar vAC localization patterns to the wild-type. These data suggest that pp28 palmitoylation at these individual cysteine residues plays a role in mature virion release, independent of its role in intracellular localization, likely by mediating interactions with viral or cellular components involved in this process.

Previous studies have shown that a pp28 myristoylation mutant was severely impaired and infectious virus could not be recovered [9]. In contrast, pp28 palmitoylation mutants, especially the TCA mutant, produced infectious extracellular virus with titers 1 and 2 logs lower compared to the wild-type virus. We showed that palmitoylation-defective pp28 remains myristoylated, whereas loss of myristoylation dramatically reduces palmitoylation, indicating that myristoylation is a prerequisite for robust palmitoylation of pp28. Furthermore, we demonstrated that myristoylation is required for membrane association, while palmitoylation is required for intracellular localization to increase the efficiency of viral release, rather than membrane association itself. The membrane association of pp28 is essential for viral assembly and maturation, processes that are directly related to viral replication. This might explain why previous studies reported non-recoverable myristoylation mutants, whereas palmitoylation-deficient viruses retain residual replication capacity.

Evolutionary conservation analysis of the pp28 N-terminal domain revealed that the palmitoylated cysteine residues are preserved across cytomegaloviruses and even other herpesvirus genera. This suggests a conserved mechanism of tegument membrane anchoring that may be critical for the coordinated assembly of herpesviruses. Palmitoylation has also been reported for HSV-1 UL11 and EBV BBLF1, both of which are membrane-associated tegument proteins. These observations support the idea that palmitoylation may serve as a conserved mechanism that facilitates tegument assembly, potentially contributing to virion egress [34].

The essential role of palmitoylation in HCMV morphogenesis highlights its potential as a target for antiviral intervention. Unlike glycoproteins, tegument proteins are often overlooked in therapeutic development. However, their central role in virion maturation and dynamic post-translational modifications make them attractive candidates. Targeting pp28 palmitoylation could destabilize the tegument structure and impair virion release without affecting viral genome replication, thereby reducing cytopathic effects and limiting viral spread. Future studies should investigate whether specific palmitoyl-transferases, such as members of the ZDHHC family, mediate pp28 acylation and whether their inhibition is feasible *in vivo*.

In conclusion, our study demonstrates that pp28 palmitoylation is essential for its stability and subcellular localization, and is required for efficient virion release. These findings underscore the broader importance of tegument lipidation in orchestrating viral morphogenesis. They also support the emerging view that tegument proteins serve structural roles and may also actively participate in viral budding. Our findings suggest that pp28 palmitoylation may influence the behavior of individual tegument proteins, as well as the function and organization of viral glycoproteins and host machinery involved in egress, such as membrane trafficking components and the ESCRT complex [35–38]. This provides new insights into the role of tegument lipidation in virion release and identifies this modification as a promising target for antiviral intervention.

## Materials and methods

### Cells, viruses, antibodies, plasmids, and reagents

HeLa cells, COS-7 cells and HEK293T cells were used for transient transfection. For virus reconstitution and propagation, human foreskin fibroblasts (HFFs) and telomerase-immortalized human fibroblasts (HFtelo) were used. HFFs and HFtelo cells were provided by Dr. P. Cresswell (Yale University). All cells were grown in DMEM (Gibco) containing 10% fetal bovine serum (HyClone) and 1% penicillin-streptomycin (HyClone). Cells were maintained at 37°C in a humidified incubator with 5% CO₂. The pHB15 BAC clone containing the HCMV AD169 genome was obtained from Dr. T. Stamminger (Ulm University Medical Center) [31]. The rpsL-neo-based recombination system used for BAC mutagenesis was provided by Dr. J. H. Ahn (Sungkyunkwan University) [39]. To generate pp28 cysteine-to-alanine mutants, we applied a two-step Red recombination approach using the AD169 BAC as a template. Recombinant BAC DNAs were transfected into HFFs using the Nucleofector Kit (Lonza) following the manufacturer’s instructions. Virus was reconstituted from transfected cells and viral DNA was extracted using the QIAamp DNA Mini Kit (Qiagen).

Monoclonal antibodies specific to HCMV proteins were used in this study. Antibodies against IE1 (UL123; clone P63-27) and pp65 (UL83; clone 28–19) were generously provided by Dr. W. J. Britt (University of Alabama at Birmingham). Commercial antibodies included an anti-myc monoclonal antibody (4A6; Merck Millipore), rabbit monoclonal antibodies against HA (Abcam), and a rat monoclonal antibody against Grp94 (Enzo Life Sciences). HRP- and fluorophore-conjugated goat anti-mouse, anti-rabbit, and anti-rat secondary antibodies were purchased from Jackson ImmunoResearch.

Expression plasmids encoding myc-tagged pp28 (wild-type and mutant forms) and UL94 were generated by PCR amplification of their respective ORFs from HCMV (AD169) genomic DNA, followed by cloning into the pcDNA3.1-Myc/His(–) vector using standard restriction-enzyme-based methods. Point mutations in pp28 were introduced via site-directed mutagenesis. All constructs were sequencing-verified to confirm the integrity of the pp28 or UL94 coding regions and the presence of the intended point mutations. Plasmids were prepared using standard endotoxin-free mini- or maxiprep procedures, quantified by NanoDrop, and transfected into HEK293T cells using Lipofectamine 2000 according to the manufacturer’s instructions.

### Acyl-RAC assay

Palmitoylation of pp28 was detected using the Badrilla CAPTUREome S-Palmitoylated Protein Kit following the manufacturer’s protocol. Briefly, cell lysates were treated with blocking and cleavage reagents to capture thioester-linked palmitoylated proteins, which were then eluted and detected via Western blotting using an anti-myc antibody.

### Triton X-114 phase separation

Triton X-114 phase partitioning was performed according to the method as described previously [12]. HEK293T cells were seeded in 6-well plates and transiently transfected with the indicated constructs for 24 h. Cells were washed once with PBS and once with TBS, collected by centrifugation, and resuspended in ice-cold TBS containing 1% Triton X-114. Samples were incubated for 1 h at 4°C. Lysates were clarified by centrifugation at 10,000 × g for 10 min at 4°C, and the resulting supernatants were layered over a cushion of 7% sucrose in TBS containing 0.1% Triton X-114. Phase separation was induced by incubation at 30°C for 10 min, followed by centrifugation at 400 × g for 4 min to allow the detergent-rich phase to collect at the bottom of the tube. The aqueous phase was removed and re-extracted by adjusting Triton X-114 to a final concentration of 1% and repeating the incubation and centrifugation steps. Detergent and aqueous fractions were mixed with SDS sample buffer, heated at 95°C for 5 min, and analyzed by SDS–PAGE and immunoblotting.

### Click-chemistry–based detection of pp28 myristoylation

Myristoylation of pp28 was assessed using the Click-iT Biotin Protein Analysis Detection Kit (Thermo Fisher Scientific, #C33372) in combination with Click-iT Myristic Acid, Azide (Thermo Fisher Scientific, #C10268). HEK293T cells were seeded in 6-well plates and transiently transfected with the indicated pp28 constructs for 24 h. Cells were labeled with azide-myristic acid according to the manufacturer’s instructions. After labeling, cells were washed in cold PBS and lysed in Brij98 lysis buffer (1% Brij98, 150 mM NaCl, 50 mM Tris-HCl, pH 7.5) supplemented with protease inhibitors. For immunoprecipitation, clarified lysates were incubated with anti-Myc antibody and Protein G magnetic beads at 4°C. Beads were washed extensively with Brij98 wash buffer, and bound proteins were eluted by incubation with 1% SDS in 50 mM Tris-HCl (pH 8.0) at 95°C for 5 min. Eluted samples were cleared by brief centrifugation and subjected to the Click-iT biotinylation reaction following the manufacturer’s protocol. Briefly, SDS-containing eluates were mixed with the Click-iT reaction buffer, CuSO₄, and biotin-alkyne reagent to catalyze the azide–alkyne cycloaddition reaction, enabling biotin tagging of azide-modified (myristoylated) pp28. Following click-labeling, samples were methanol-chloroform precipitated, resuspended in SDS sample buffer, and resolved by SDS-PAGE. Biotinylated pp28 was detected by immunoblotting using streptavidin-HRP. Total pp28 expression levels were assessed in parallel by immunoblotting with anti-Myc antibody.

### Prediction of palmitoylation sites and sequence conservation analysis

Palmitoylation site prediction was performed using the CSS-Palm 4.0 algorithm [26]. For sequence conservation analysis, protein sequences of pp28 orthologs from various cytomegaloviruses and other herpesviruses were aligned using Clustal Omega [40].

### Proteasome and lysosome inhibition assays

To investigate whether pp28 protein stability is influenced by proteasomal or lysosomal degradation, HEK293T cells were transfected with expression plasmids encoding either wild-type or mutant forms of pp28. At 24 hpi (hours post-transfection), cells were treated with each inhibitor for an additional 24 h. Proteasome function was blocked using MG132 (10 µM), epoxomicin (1 µM), or DBeQ (10 µM). Lysosomal degradation was inhibited using leupeptin (100 µM), chloroquine (50 µM), or bafilomycin A1 (100 nM). As a control, cells were treated with DMSO. After inhibitor treatment, cells were washed with cold PBS and lysed in Cell Lysis Buffer (Cell Signaling Technology, #9803) prepared at a 1 × working concentration with the addition of protease inhibitors. Lysates were clarified by centrifugation and subjected to SDS-PAGE followed by immunoblotting to evaluate pp28 protein levels under each condition.

### RNA extraction and quantitative real-time PCR

Total RNA was isolated from cultured cells using the Total RNA Purification Kit for cultured cells (Favorgen) according to the manufacturer’s protocol. First-strand cDNA was generated from 1 μg of total RNA using the PrimeScript RT reagent kit (TaKaRa Bio). Quantitative real-time PCR (qRT-PCR) was carried out using the TB Green Fast qPCR reagent kit (TaKaRa Bio) to assess gene expression levels. The primers used for PCR are listed (S1 Table). Each reaction was performed in technical triplicates. Relative transcript levels were calculated using the comparative threshold cycle (2^–ΔΔCT^) method, with β-actin serving as the internal normalization control. Data were derived from three independent biological replicates. Statistical comparisons of gene expression are presented with corresponding *P* values in the figure panels.

The viral genome copy number was also quantified by real-time PCR as described previously [10]. Viral DNA were isolated by QIAamp DNA blood kit (Qiagen). The primers, which amplify a segment of the HCMV UL55 gene, used for PCR are listed (S1 Table).

### Immunoblot analysis

Cells were lysed using Cell Lysis Buffer (Cell Signaling Technology, #9803) supplemented with protease inhibitors. Lysates were clarified by centrifugation, and total protein concentrations were measured using the bicinchoninic acid (BCA) assay (Thermo Fisher Scientific). Proteins were transferred to polyvinylidene fluoride (PVDF) membranes (Merck Millipore) and membranes were blocked with 5% skim milk and 0.05% Tween-20 in phosphate-buffered saline (PBS). Blots were incubated with primary antibodies followed by horseradish peroxidase-conjugated secondary antibodies, and signal was detected using enhanced chemiluminescence reagents (Thermo Fisher Scientific). Grp94 served as a loading control.

### Immunofluorescence

Cells grown on coverslips were fixed with 4% paraformaldehyde and then permeabilized using 0.2% Triton X-100. After permeabilization, cells were blocked with 5% bovine serum albumin in PBS. Primary antibodies against myc, ERGIC-53 and UL94 were applied to the samples and subsequently detected using Alexa Fluor-conjugated secondary antibodies from Thermo Fisher. Nuclei were stained with DAPI. Confocal images were captured using a Zeiss LSM700 microscope equipped with a 63 × objective lens.

### Electron microscopy

HFFs were infected with HCMV wild-type or recombinant HCMV expressing pp28 TCA at an moi of 1 and examined by electron microscopy at 7 dpi as described previously [41]. Briefly, the cells were fixed with 2.5% glutaraldehyde and post-fixed with 1% osmium tetroxide. Cells were *en bloc* stained with 2% uranyl acetate, dehydrated, infiltrated, and embedded in Epon. Sixty nanometer sections were stained with lead citrate and examined using a JEM-1011 (JEOL) electron microscope.

### Bacmid mutagenesis

Cysteine-to-alanine point mutations in pp28 (UL99) were introduced into the HCMV (AD169)-BAC backbone using a two-step Red recombination strategy with an rpsL-neo counter-selection cassette, as previously described. An rpsL-neo cassette flanked by 50-nucleotide homology arms targeting the 5′ region of UL99 was PCR-amplified and introduced into *E. coli* DH10B cells harboring the BAC by electroporation, and recombinant clones were selected on kanamycin-containing LB agar plates. In the second recombination step, the cassette was replaced by annealed oligonucleotide duplexes encoding the desired cysteine-to-alanine mutations using the same homology arms, and positive recombinants were selected on streptomycin. The following mutants were generated: C6A, C10A, C11A, C6A/C10A, C10A/C11A, C6A/C10A/C11A (TCA), and a revertant in which the wild-type sequence was restored. The primer set for PCR amplification of pp28 mutants is listed (S2 Table). PCR and sequencing were performed on the modified UL99 regions to confirm the presence of the desired mutations in the recombinant BAC clones.

### Virus entry assays

Virus entry was evaluated by assessing the expression of the immediate early protein IE1 using immunofluorescence staining and immunoblot analysis, based on previously described protocols [42]. For immunofluorescence, human fibroblasts were seeded onto sterile glass coverslips placed in 24-well plates and cultured overnight. On the following day, cells were pre-chilled at 4°C for 15 min and incubated with HCMV at 4°C for 90 min to allow virus binding. The cultures were then transferred to 37°C for 30 min to facilitate viral penetration. To inactivate uninternalized virus, cells were treated with low-pH citrate buffer for 1 min and subsequently washed twice with complete medium. Infected cells were incubated at 37°C for an additional 24 h. Cells were then fixed with 4% paraformaldehyde in PBS for 20 min at room temperature, permeabilized with 0.1% Triton X-100 in PBS, and stained with an anti-IE1 monoclonal antibody. A fluorophore-conjugated secondary antibody and DAPI were used to visualize IE1 expression and nuclear DNA, respectively. The proportion of IE1-positive cells was quantified by fluorescence microscopy. For immunoblot analysis, cells were harvested at 15–24 hpi, pelleted by centrifugation, and resuspended in 3 × Laemmli sample buffer. Proteins were separated by SDS-PAGE and transferred to nitrocellulose membranes. Blots were incubated with anti-IE1 antibody followed by horseradish peroxidase (HRP)–conjugated secondary antibody. Protein bands were visualized using chemiluminescence detection reagents.

### Virus titration assay

To quantify infectious HCMV particles, both extracellular and intracellular virus titers were determined by immunofluorescence-based titration using IE1 staining [32]. HFFs were seeded in 96-well plates and infected in duplicate with 10-fold serial dilutions of virus-containing supernatants (extracellular titers) or cell lysates (intracellular titers) harvested at the indicated time points post-infection. At 24 hpi, cells were fixed in 4% paraformaldehyde for 30 minutes, permeabilized with 0.1% Triton X-100, and incubated with a monoclonal antibody specific for the HCMV IE1. Infected foci were visualized by fluorescence microscopy and quantified to calculate infectious units per milliliter. Endpoint titers were calculated based on the highest dilution that produced detectable IE1-positive cells.

Plaque forming assay (PFA) was also used for virus titration. 1.8 × 10^5^ of human foreskin fibroblast (HFF) cells were seeded in 12-well plates and cultured overnight. The growth medium was removed and the cells were washed with DMEM. Serial dilutions of virus-containing supernatants from HCMV-infected HFFs were prepared in DMEM and added to the cells in duplicate. The plates were incubated for 2 hours at 37°C. After incubation, the inoculum was aspirated, and the cells were washed gently with DMEM. An equal-volume mixture of 2% SeaPlaque agarose (Lonza, 50101) and 2 × DMEM (Gibco, 12800–017) supplemented with 10% FBS and 1% penicillin-streptomycin was prepared and overlaid onto the cells. After 8 days of incubation, the cells were fixed overnight at 4°C with 4% paraformaldehyde (Biosesang, PC2031-050-00) and stained with 0.5% Gentian violet (Duksan, 754). The plaques were enumerated manually using a microscope, and viral titers were calculated based on the plaque numbers.

### Statistical analysis

The data are presented as mean ± standard error of the mean (SEM). Statistical analyses were performed using GraphPad Prism software (version 9.51). For comparisons between two groups, an unpaired two-tailed Student’s *t*-test was used. For comparisons among multiple groups, one-way ANOVA with Dunnett’s multiple-comparison test was used. *P* values < 0.05 were considered to be statistically significant.

## Supporting information

S1 Fig Alanine substitution of Cys67 and Cys71 does not impair pp28 palmitoylation. HEK293T cells were transiently transfected with wild-type pp28-myc or C-terminal cysteine-to-alanine mutants (C67A or C67A/C71A). Palmitoylated pp28 (Pal-pp28) was isolated by Acyl-RAC and detected by immunoblotting. Unlike the N-terminal cysteine mutants, substitution of Cys67 or Cys71 did not reduce pp28 palmitoylation. Bar graphs represent palmitoylation levels normalized to input pp28.
(TIF)

S2 Fig Palmitoylation-defective pp28 remains myristoylated, but myristoylation loss severely impairs palmitoylation. (A) HEK293T cells expressing the indicated pp28 constructs were treated with DMSO or 2-BP (50 µM) for 24 h or (B) untreated. Myristoylated pp28 was detected using Click chemistry–based labeling, and total pp28 was analyzed by immunoblotting. Bar graphs represent myristoylation levels normalized to input pp28. (C) HEK293T cells expressing the indicated pp28 constructs were treated with DMSO or MG132 (5 µM) for 20 h and subjected to Acyl-RAC to detect palmitoylated pp28. Total pp28 and Grp94 are shown for input samples. Bar graphs represent palmitoylation levels normalized to input pp28.
(TIF)

S3 Fig Palmitoylation-deficient pp28 (TCA) misdirects UL94 localization. Confocal immunofluorescence analysis of 293T cells co-transfected with UL94-HA and either wild-type pp28-myc or the palmitoylation-deficient TCA mutant. Cells were stained antibodies against myc (pp28), HA (UL94), and ERGIC-53. Nuclei were counterstained with DAPI (blue). Scale bars, 10 μm.
(TIF)

S4 Fig Comparison of extracellular and intracellular viral titers by plaque forming assay. (A) Extracellular viral titers were determined by the plaque forming assay in HFF cells infected with wild-type, TCA, or revertant viruses. Supernatants were collected at the indicated dpi, and infectious units were quantified as PFU/mL. Note that the 3 dpi values for the TCA mutant virus are near the limit of detection. (B) Intracellular viral titers were measured by the plaque forming assay following cell-associated virus release. Infected cells were harvested at the indicated dpi, subjected to freeze–thaw cycles, and titrated by plaque formation in HFF cells. Data are presented as PFU/mL. The right panels show bar graphs depicting virus titers at 3, 5, 7, and 9 dpi. Data are presented as means ± SEM. Statistical analysis was performed using one-way ANOVA with Dunnett’s multiple-comparison test. *P < 0.05; **P < 0.01; ***P < 0.001.
(TIF)

S5 Fig pp28 palmitoylation affects viral release, not intracellular virion envelopment. (A) Transmission electron micrographs of HFFs infected with wild-type or recombinant HCMV mutant encoding pp28 TCA. Cells were infected at an moi of 1 and processed for EM at 7 dpi. Multiple frames from each sample were imaged and photographed. Particles from a representative cell are shown. White and black arrows indicate non-enveloped particles and enveloped particles, respectively. Nu, nucleus; Cyto, cytoplasm. Scale bar, 1 μm. (B) The numbers of enveloped particles were counted in each frame and calculated as a ratio of enveloped particles to total particles in the cytoplasm of infected cells. The graphs indicate the mean percent of the enveloped particles per total particles in 25–35 frames (from >10 cells of each sample) ± SEM from two independent experiments. Statistical analysis was performed using a *t*-*t*est. (C) HFFs were infected with the wild-type, the TCA mutant, and the revertant (TCA-REV) at an moi of 1. Viral genome copy numbers in viral particles harvested from supernatants at 7 dpi were measured by real-time PCR. The infectious particle numbers were determined by immunofluorescence titration assays. The graph depicts the ratio of the viral genome copy numbers to the infectious particle numbers. Data are presented as means ± SEM. Statistical analysis was performed using one-way ANOVA with Dunnett’s multiple-comparison test. (D) Extracellular and intracellular viral titers were determined by the plaque forming assay in HFF cells infected with wild-type, TCA, or revertant viruses at 13 dpi. Data are presented as means ± SEM. Statistical analysis was performed using one-way ANOVA with Dunnett’s multiple-comparison test. **P < 0.01; ***P < 0.001.
(TIF)

S6 Fig pp28 palmitoylation does not affect HCMV entry efficiency. (A) Immunoblot analysis of IE1 protein levels in HFFs infected with wild-type, palmitoylation-deficient (TCA), or revertant (TCA-REV) HCMV at an moi of 2. Cells were harvested at 6 hpi. A lower moi (0.2) was included as a reference control. (B) Fluorescence-based HCMV entry assay. HFFs were infected with wild-type, TCA, or TCA-REV viruses at an moi of 2 and fixed 6 hpi. Infected cells were immunostained for IE1 (green), and the number of IE1-positive cells per field was quantified from fluorescence images. Scale bars, 100 μm. The right panel shows bar graphs illustrating the number of IE1-positive cells per milliliter of infectious virus at 6 hpi. Data are presented as means ± SEM from two independent experiments. Statistical analysis was performed using one-way ANOVA with Dunnett’s multiple-comparison test.
(TIF)

S1 Table Primer sequences used for quantitative real-time PCR. (DOCX)

S2 Table PCR primer sequences used for bacmid mutagenesis. (DOCX)

S1 File Source data. (XLSX)
