# Supplementary figures and images for: Palmitoylation of the human cytomegalovirus tegument protein pp28 facilitates virus release
Source: PLoS Pathog. 2026 Jan 22;22(1):e1013894. doi: 10.1371/journal.ppat.1013894 (PMC12851460; doi:10.1371/journal.ppat.1013894)

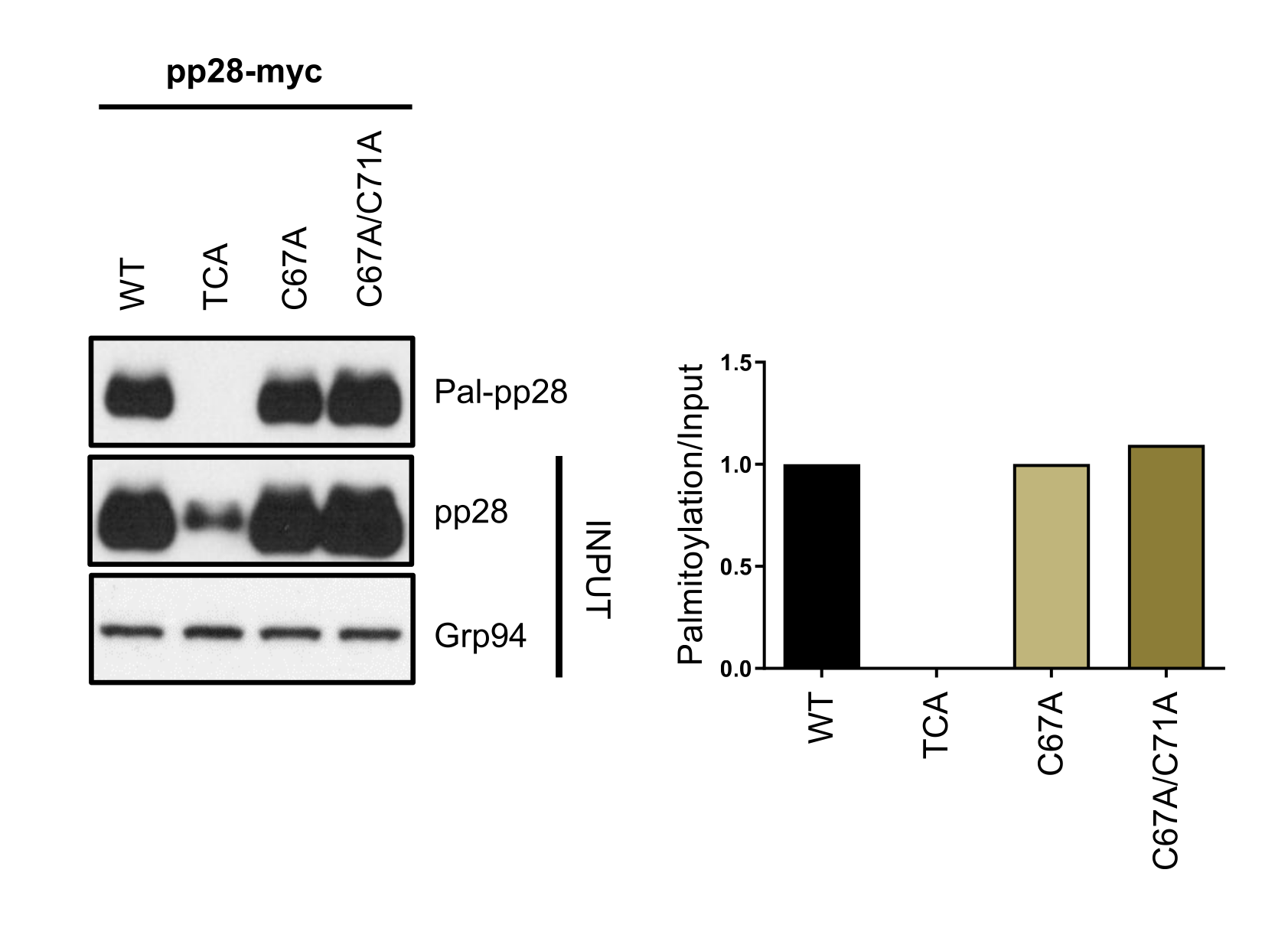

Supplement: S1 Fig — HEK293T cells were transiently transfected with wild-type pp28-myc or C-terminal cysteine-to-alanine mutants (C67A or C67A/C71A). Palmitoylated pp28 (Pal-pp28) was isolated by Acyl-RAC and detected by immunoblotting. Unlike the N-terminal cysteine mutants, substitution of Cys67 or Cys71 did not reduce pp28 palmitoylation. Bar graphs represent palmitoylation levels normalized to input pp28. (TIF) [file ppat.1013894.s001.tif]

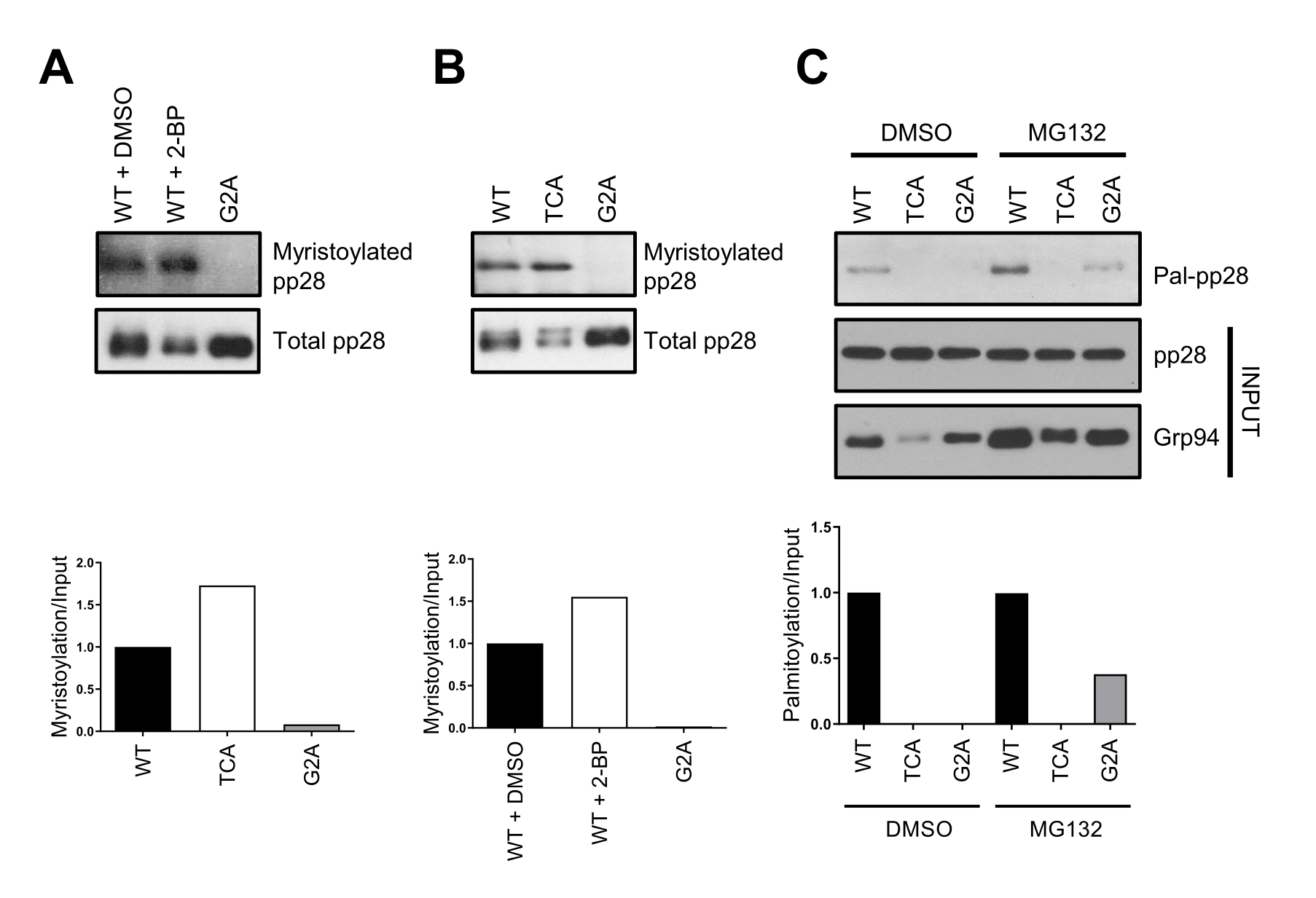

Supplement: S2 Fig — (A) HEK293T cells expressing the indicated pp28 constructs were treated with DMSO or 2-BP (50 µM) for 24 h or (B) untreated. Myristoylated pp28 was detected using Click chemistry–based labeling, and total pp28 was analyzed by immunoblotting. Bar graphs represent myristoylation levels normalized to input pp28. (C) HEK293T cells expressing the indicated pp28 constructs were treated with DMSO or MG132 (5 µM) for 20 h and subjected to Acyl-RAC to detect palmitoylated pp28. Total pp28 and Grp94 are shown for input samples. Bar graphs represent palmitoylation levels normalized to input pp28. (TIF) [file ppat.1013894.s002.tif]

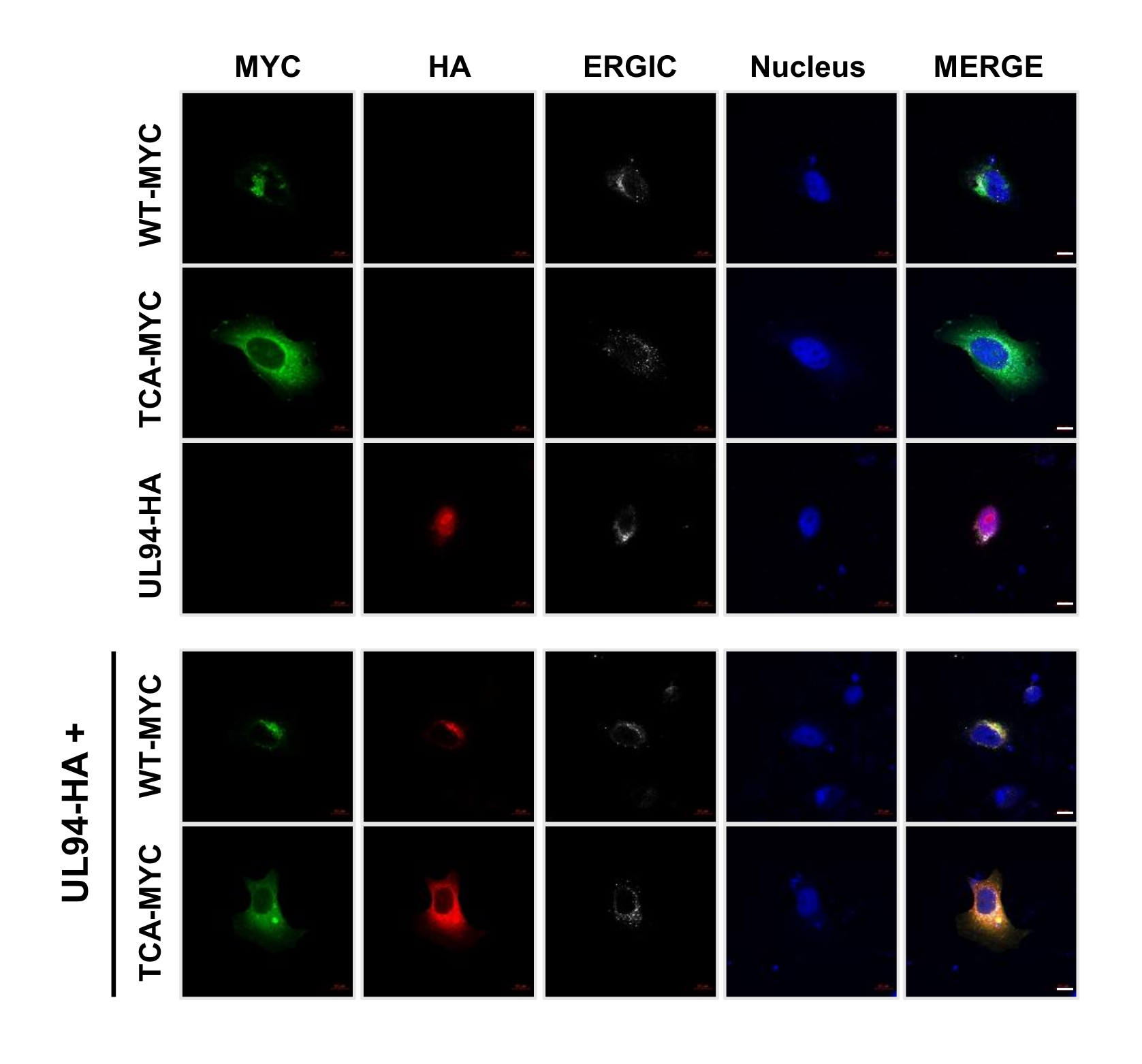

Supplement: S3 Fig — Confocal immunofluorescence analysis of 293T cells co-transfected with UL94-HA and either wild-type pp28-myc or the palmitoylation-deficient TCA mutant. Cells were stained antibodies against myc (pp28), HA (UL94), and ERGIC-53. Nuclei were counterstained with DAPI (blue). Scale bars, 10 μm. (TIF) [file ppat.1013894.s003.tif]

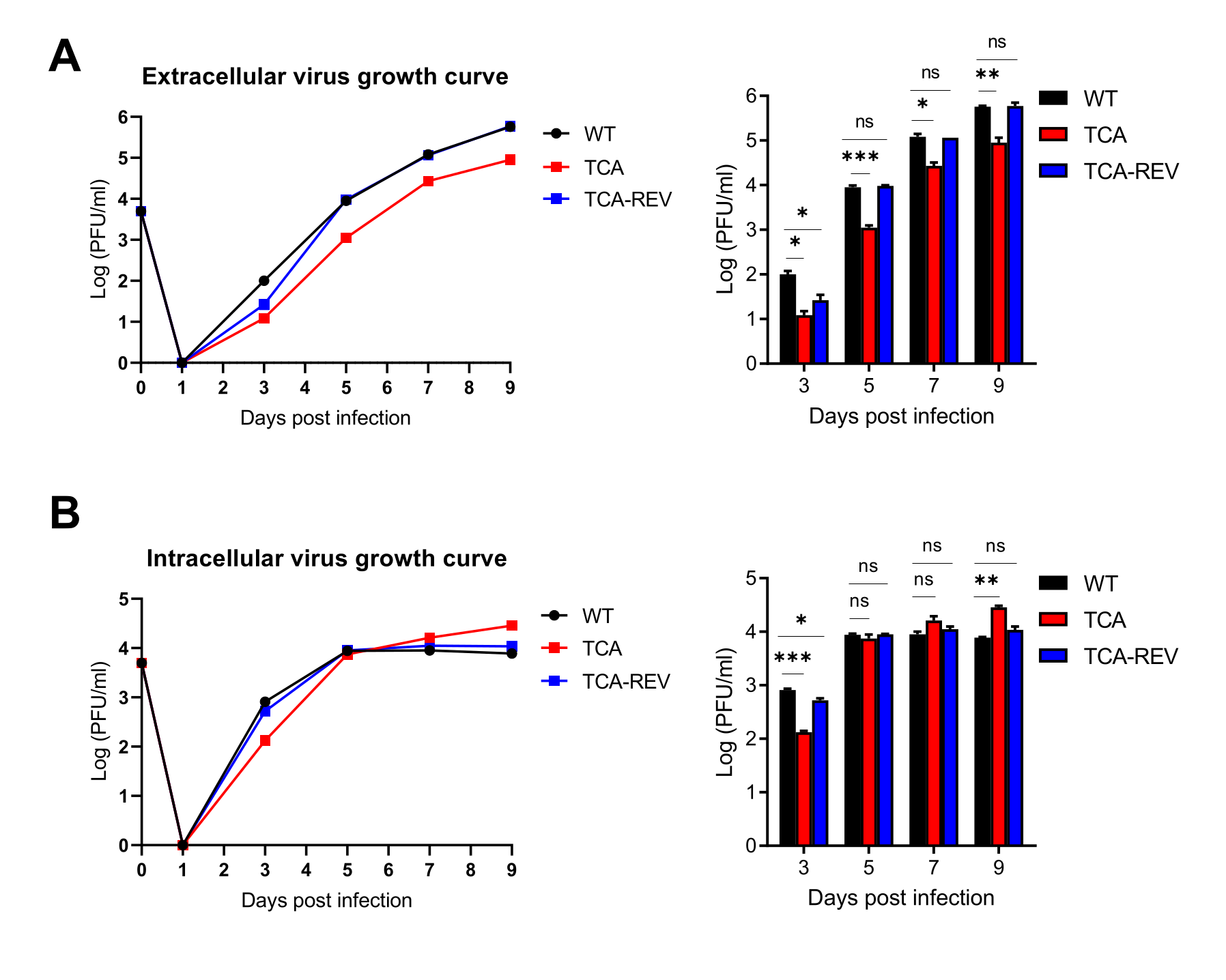

Supplement: S4 Fig — (A) Extracellular viral titers were determined by the plaque forming assay in HFF cells infected with wild-type, TCA, or revertant viruses. Supernatants were collected at the indicated dpi, and infectious units were quantified as PFU/mL. Note that the 3 dpi values for the TCA mutant virus are near the limit of detection. (B) Intracellular viral titers were measured by the plaque forming assay following cell-associated virus release. Infected cells were harvested at the indicated dpi, subjected to freeze–thaw cycles, and titrated by plaque formation in HFF cells. Data are presented as PFU/mL. The right panels show bar graphs depicting virus titers at 3, 5, 7, and 9 dpi. Data are presented as means ± SEM. Statistical analysis was performed using one-way ANOVA with Dunnett’s multiple-comparison test. *P < 0.05; **P < 0.01; ***P < 0.001. (TIF) [file ppat.1013894.s004.tif]

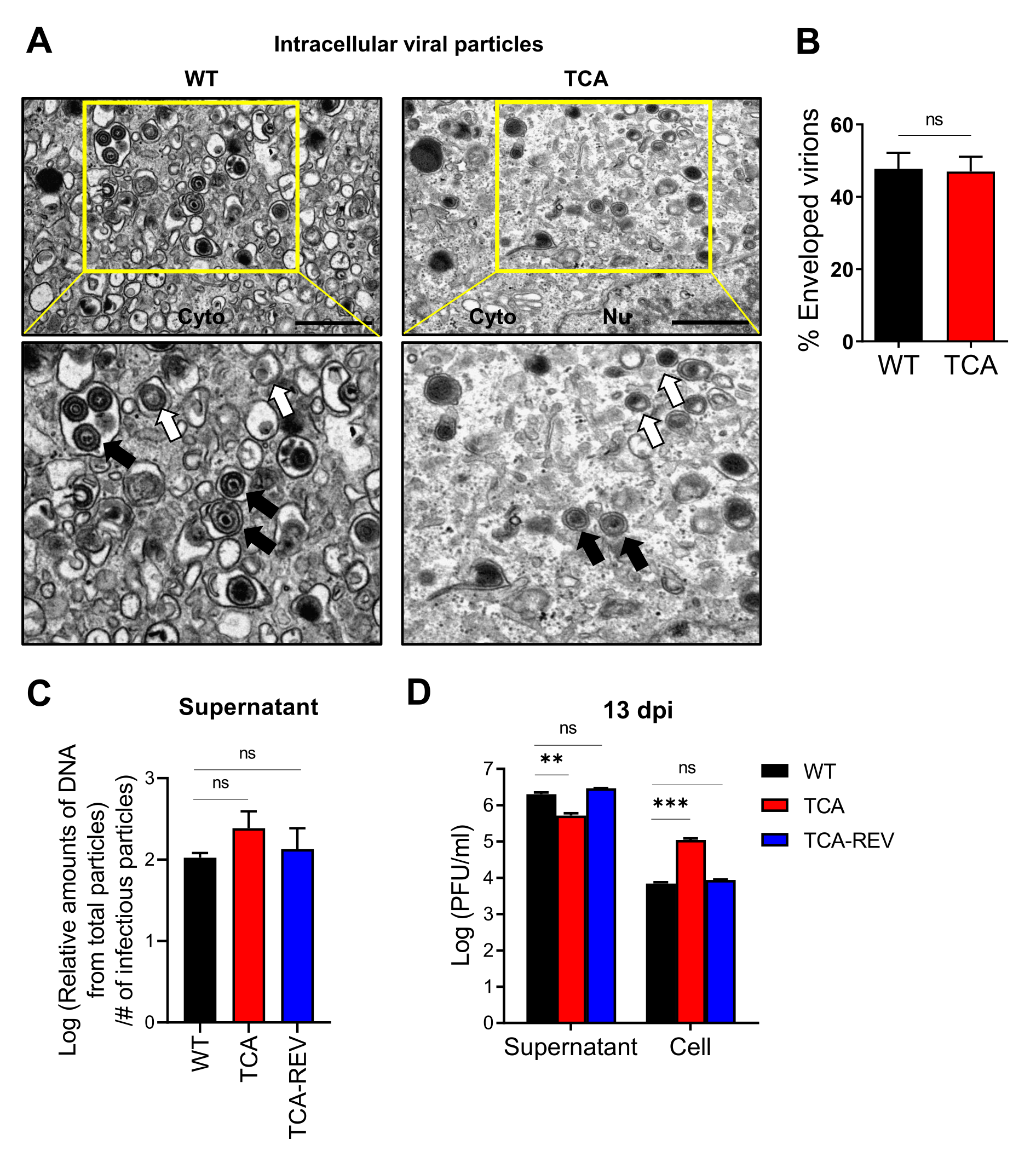

Supplement: S5 Fig — (A) Transmission electron micrographs of HFFs infected with wild-type or recombinant HCMV mutant encoding pp28 TCA. Cells were infected at an moi of 1 and processed for EM at 7 dpi. Multiple frames from each sample were imaged and photographed. Particles from a representative cell are shown. White and black arrows indicate non-enveloped particles and enveloped particles, respectively. Nu, nucleus; Cyto, cytoplasm. Scale bar, 1 μm. (B) The numbers of enveloped particles were counted in each frame and calculated as a ratio of enveloped particles to total particles in the cytoplasm of infected cells. The graphs indicate the mean percent of the enveloped particles per total particles in 25–35 frames (from >10 cells of each sample) ± SEM from two independent experiments. Statistical analysis was performed using a t-test. (C) HFFs were infected with the wild-type, the TCA mutant, and the revertant (TCA-REV) at an moi of 1. Viral genome copy numbers in viral particles harvested from supernatants at 7 dpi were measured by real-time PCR. The infectious particle numbers were determined by immunofluorescence titration assays. The graph depicts the ratio of the viral genome copy numbers to the infectious particle numbers. Data are presented as means ± SEM. Statistical analysis was performed using one-way ANOVA with Dunnett’s multiple-comparison test. (D) Extracellular and intracellular viral titers were determined by the plaque forming assay in HFF cells infected with wild-type, TCA, or revertant viruses at 13 dpi. Data are presented as means ± SEM. Statistical analysis was performed using one-way ANOVA with Dunnett’s multiple-comparison test. **P < 0.01; ***P < 0.001. (TIF) [file ppat.1013894.s005.tif]

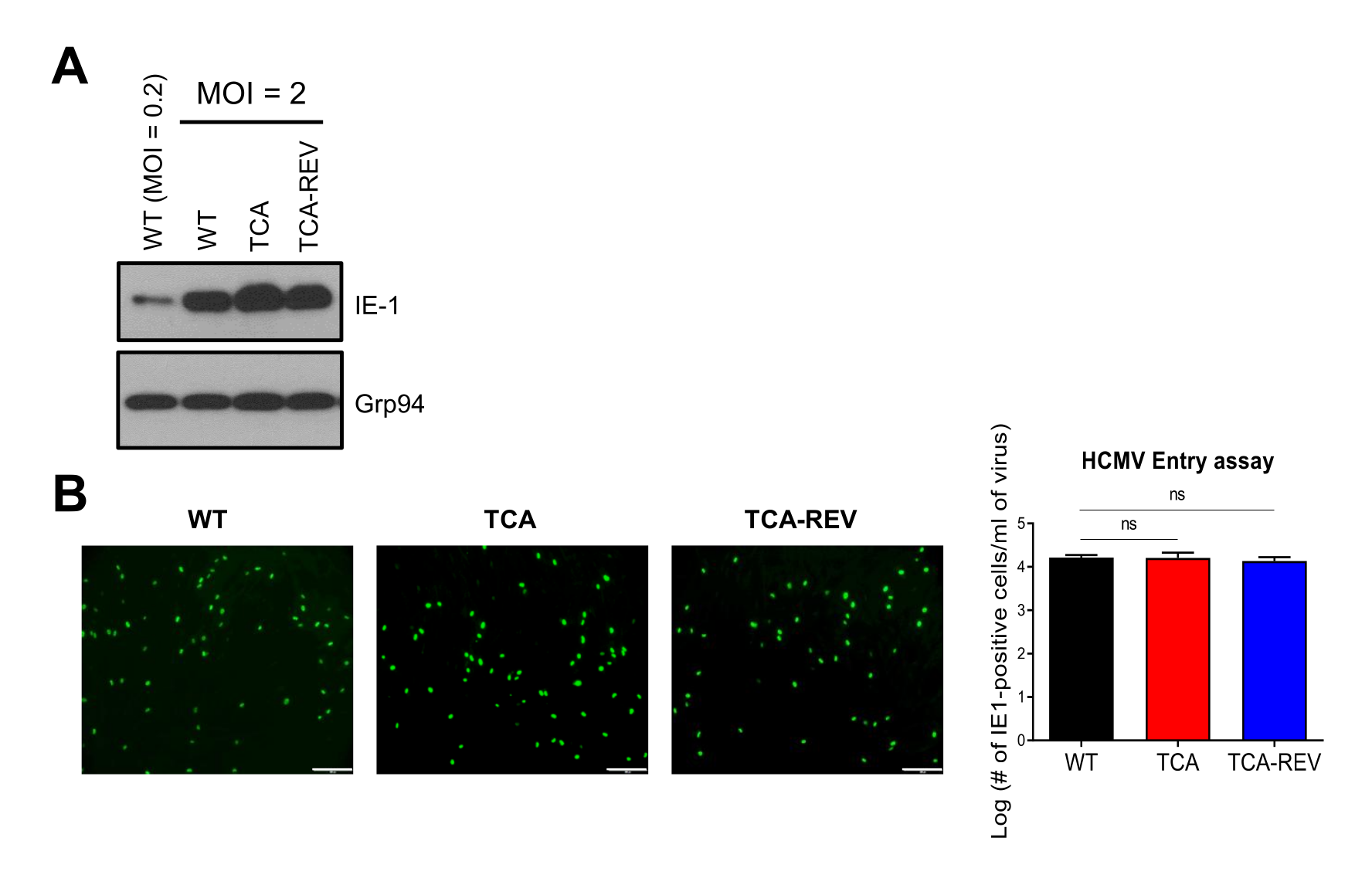

Supplement: S6 Fig — (A) Immunoblot analysis of IE1 protein levels in HFFs infected with wild-type, palmitoylation-deficient (TCA), or revertant (TCA-REV) HCMV at an moi of 2. Cells were harvested at 6 hpi. A lower moi (0.2) was included as a reference control. (B) Fluorescence-based HCMV entry assay. HFFs were infected with wild-type, TCA, or TCA-REV viruses at an moi of 2 and fixed 6 hpi. Infected cells were immunostained for IE1 (green), and the number of IE1-positive cells per field was quantified from fluorescence images. Scale bars, 100 μm. The right panel shows bar graphs illustrating the number of IE1-positive cells per milliliter of infectious virus at 6 hpi. Data are presented as means ± SEM from two independent experiments. Statistical analysis was performed using one-way ANOVA with Dunnett’s multiple-comparison test. (TIF) [file ppat.1013894.s006.tif]
