# Supplementary material for: Palmitoylation of the human cytomegalovirus tegument protein pp28 facilitates virus release
Source: PLoS Pathog. 2026 Jan 22;22(1):e1013894. doi: 10.1371/journal.ppat.1013894 (PMC12851460; doi:10.1371/journal.ppat.1013894)
Supplement: S1 Table — (DOCX) [file ppat.1013894.s007.docx]

**S1 Table.** Primer sequences used for quantitative real-time PCR.

| **Primer name** | **Forward (5′ → 3′)** | **Reverse (5′ → 3′)** |
| --- | --- | --- |
| UL99 | CGGGGGAAACGACAGTAGTA | CTGATGGTGGTGACGTTTTG |
| UL55 | ACGTGAAGGAATCGCCAGGA | AGTTCCAGTACCCTGAAGTC |
| β-actin | GCTCCGGCATGTGCAA | AGGATCTTCATGAGGTAGT |
