# Supplementary material for: Palmitoylation of the human cytomegalovirus tegument protein pp28 facilitates virus release
Source: PLoS Pathog. 2026 Jan 22;22(1):e1013894. doi: 10.1371/journal.ppat.1013894 (PMC12851460; doi:10.1371/journal.ppat.1013894)
Supplement: S2 Table — (DOCX) [file ppat.1013894.s008.docx]

**S2 Table.** PCR primer sequences used for bacmid mutagenesis.

| **Primer name** | **Forward (5′ → 3′)** | **Reverse (5′ → 3′)** |
| --- | --- | --- |
| rpsL-neo-set | AGTCGCGACCTGTCCCGCAAGACGAACCTACCGATATGGGTGCCG AACTCGGCCTGGTGATGATGGCGGGATCG | TGGCGACCCAGAGCATCTTTCAGGGGCTCACCGGGCGTGGTACCGAACTCTCAGAAGAACTCGTCAAGAAGGCG |
| pp28 | AGTCGCGACCTGTCCCGCAAGACGAACCTACCGATATGGGTGCCGAACTC | TGGCGACCCAGAGCATCTTTCAGG |
